# Apigenin Protects the Brain against Ischemia/Reperfusion Injury via Caveolin-1/VEGF In Vitro and In Vivo

**DOI:** 10.1155/2018/7017204

**Published:** 2018-12-03

**Authors:** Qiongyi Pang, Yun Zhao, Xiang Chen, Kaiyi Zhao, Qiongxiang Zhai, Fengxia Tu

**Affiliations:** ^1^The Second Affiliated Hospital and Yuying Children's Hospital of Wenzhou Medical University, 109 Xueyuan Western Road, Wenzhou, Zhejiang Province 325027, China; ^2^Department of Pediatrics, Guangdong General Hospital, Guangdong Academy of Medical Sciences, 106 Zhongshan Second Road, Guangzhou, Guangdong Province 510080, China

## Abstract

Apigenin is a natural flavonoid found in several dietary plant foods as vegetables and fruits. To investigate potential anti-ischemia/reperfusion injury properties of apigenin in vitro, cell proliferation assay, tube formation, cell migration, apoptosis, and autophagy were performed in human brain microvascular endothelial cells (HBMVECs) after oxygen-glucose deprivation/reoxygenation (OGD/R). The effect of apigenin was also explored in rats after middle cerebral artery occlusion/reperfusion (MCAO/R) via neurobehavioral scores, pathological examination, and measurement of markers involved in ischemia/reperfusion injury. Data in vitro indicated that apigenin could prompt cell proliferation, tube formation, and cell migration while inhibiting apoptosis and autophagy by affecting Caveolin-1/VEGF, Bcl-2, Caspase-3, Beclin-1, and mTOR expression. Results in vivo showed that apigenin significantly reduced neurobehavioral scores and volume of cerebral infarction while prompting vascular endothelial cell proliferation by upregulating VEGFR2/CD34 double-labeling endothelial progenitor cell (EPC) number and affecting Caveolin-1, VEGF, and eNOS expression in brain tissue of MCAO/R rats. All the data suggested that apigenin may be protective for the brain against ischemia/reperfusion injury by alleviating apoptosis and autophagy, promoting cell proliferation in HBMVECs of OGD/R, and attenuating brain damage and improved neurological function in rats of MCAO/R through the Caveolin-1/VEGF pathway.

## 1. Introduction

Ischemia such as stroke, myocardial infarction, and peripheral vascular disease is one of the most common causes of disability and death worldwide [1]. The extent of tissue injury relates directly to the extent of blood flow reduction and to the length of the ischemic period. The brain exhibits the highest sensitivity to ischemia of all the body organs because it has the highest metabolic activity and has significantly lower levels of protective antioxidant enzymes [2, 3]. Focal cerebral ischemia, also known as ischemic stroke, arising in a specific vascular territory secondary to thromboembolic or atherothrombotic vasoocclusion, is the most common clinical presentation of ischemia/reperfusion of the brain. The brain requires constant delivery of glucose to maintain its metabolic demands as an absolute requirement. Ischemia/reperfusion of tissues is often associated with microvascular injury.

Apigenin is one of many natural flavonoids found in vegetables and fruits, such as celery, parsley, onion, tea, and grapefruit. Flavonoids can act as antioxidants to protect biomolecules from oxidative damage through free radical-mediated reactions [4]. It has been reported that apigenin plays a protective role in diseases associated with the oxidative process, such as cardiovascular and neurological disorders. Apigenin alleviates myocardial toxicity by modulating oxidative stress and inflammation in lipopolysaccharide- (LPS-) induced myocardial injury model [5]. Reactive oxygen species (ROS) and malondialdehyde (MDA) were significantly decreased by apigenin in acrylonitrile-induced subchronic sperm injury in rats [6]. Apigenin pretreatment can protect against renal ischemia/reperfusion via the activation of the JAK2/STAT3 signaling pathway [7]. Siddique and Jyoti reported that apigenin is potent in increasing the life span by reducing the oxidative stress as well as apoptosis in the model of Parkinson's disease [8]. Caveolin-1 as an iconic structural protein of caveolae, affects tumor growth and ischemia by regulating embryonic vessel development, normal tissue growth, cellular signal transduction, and molecular transport [9, 10]. VEGF (vascular endothelial growth factor) associates with angiogenesis protecting neurons from ischemic insults and promoting neurogenesis after cerebral ischemic injury [11–14].

We have demonstrated that Caveolin-1 decreased cerebral infarct volume, facilitated angiogenesis and neurogenesis, and promoted neurological recovery by upregulating the Caveolin-1/VEGF signaling pathway in a rat model of middle cerebral artery occlusion (MCAO) in our previous study [15–17]. Apigenin ameliorates poststroke cognitive deficits through alteration of histone acetylation-mediated neurochemicals in vivo in another study [18]. Based on our previous study, we explored the effect of apigenin on the Caveolin-1/VEGF signaling pathway in an oxygen-glucose deprivation/reoxygenation (OGD/R) model of human brain microvascular endothelial cells (HBMVECs) and MCAO rat model.

## 2. Materials and Methods

### 2.1. Cell and Cell Culture

Human brain microvascular endothelial cells (HBMVECs) were purchased from Angio-Proteomie (MA, USA). The cells were cultured in an endothelial cell medium (ECM) (ScienCell, CA), containing endothelial cell growth supplement (ECGS) and 5% fetal bovine serum (FBS). Cells were incubated with an atmosphere of 5% CO_2_ and 95% air. The glucose-free ECM was also provided by ScienCell Research Laboratories Inc.

### 2.2. Model of Oxygen-Glucose Deprivation/Reoxygenation (OGD/R) and Treatment with Drugs In Vitro

HBMVECs were exposed to hypoxic conditions of oxygen deprivation, 1% O_2_ + 5% CO_2_ + 94% N_2_ for 4 h, 6 h, 8 h, and 12 h in glucose-free culture. After hypoxia, the cells were reoxygenated for 24 h or 48 h in a complete medium. Apigenin (Sigma-Aldrich, MO, USA) with concentrations of 2.5, 5, and 10 *μ*M was added to the cells prior to hypoxia.

### 2.3. Lentivirus and Lentivirus Infection

Caveolin-1 knockdown lentivirus was provided by Shanghai R&S Biotechnology Co. Ltd. Before lentivirus infection, HBMVECs were seeded into 10 cm dishes (5 × 10^6^ cells/dish) and incubated for 12 h. Then the lentivirus was added into dishes with a multiplicity of infection (MOI) of 10 to infect cells. When the efficiency was about 90% after infection detected by fluorescence microscopy analysis of GFP, HBMVECs were used for experiments.

### 2.4. Cell Viability Assay

5 × 10^3^ HBMVECs/well were plated in 96-well plates. After 24 h of culture, cells were under hypoxia-oxygenation for a designed time or treated with different concentrations of apigenin in glucose-free culture followed by reoxygenation and cultured in normal conditions. Cell viability was measured by Cell Counting Kit-8 (CCK8) (Dojindo, Japan). After treatment with 20 *μ*L of CCK8 solution at 37°C for 2 h, the Thermo Multiskan MK3 (Thermo, MA, USA) microplate reader was used to quantify the formazan products by measuring the absorbance at 450 nm.

### 2.5. Real-Time Quantitative PCR (qRT-PCR)

After treatment, HBMVECs were collected, of which total RNA was extracted using TRIzol reagent (Invitrogen, USA) according to the manufacturer's protocol. Total RNA of infarct tissue of the brain was extracted similar to HBMVECs. Total RNA was reverse-transcribed using the One-Step RT-qPCR Kit (Sangon Biotech, China) and then amplified with PCR primers on the iQ5 Real-Time Quantitative PCR (BioRad, USA). Real-time PCR primers for Caveolin-1, mTOR, BCL-2, Beclin-1, Caspase-3, and VEGF are shown in Table 1. The PCR cycling profile included predenaturing at 95°C for 3 min, 40 cycles of denaturing at 95°C for 15 sec, annealing at 60°C for 20 sec, and extension at 72°C for 30 sec. The 2^−ΔΔCt^ method (ΔΔCt = (C_target mRNA_ − Ct_ACTB_) − (control − Ct_ACTB_)) was used to quantify the relative expression of the target mRNA. Relative expression of mRNA was normalized to the control. ACTB was chosen as the internal control.

### 2.6. Western Blotting

After OGD/R (middle cerebral artery occlusion/reperfusion MCAO/R) and treatment with apigenin, HBMVECs and infarct brain tissue were harvested and lysed in a SDS-PAGE-loading buffer, then centrifuged for 2 min at 11,000 × g at 4°C to collect the supernatant. The supernatant was resolved by 10% SDS-PAGE gel and transferred electrophoretically onto PVDF (Millipore, Shanghai, China). After being blocked by 5% skim milk in Tris-buffered saline containing TBST, the membranes were incubated with primary antibodies against *β*-tubulin (Sungene Biotech, China), Caveolin-1 (Santa Cruz Biotechnology, USA), Bcl-2 (Abcam, UK), VEGF (CST, USA), eNOS (Abcam, UK), cleaved Caspase3 (CST, USA), Beclin-1 (CST), and mTOR (CST, USA) and were incubated overnight at 4°C. Then the membranes were washed and incubated with secondary antibodies (Sungene Biotech, China). Blots were then incubated and visualized with enhanced chemiluminescence (Thermo Scientific, Shanghai, China) by an automatic chemiluminescence image analysis system (Tanon, China). The results were normalized to *β*-tubulin to correct for loading.

### 2.7. Migration Assay

Transwell was used to measure cell migration. Briefly, 1 × 10^4^ HBMVECs were seeded onto Transwell chambers with/without treatment with apigenin accompanied with OGD/R, followed by being placed in a 24-well plate containing the complete medium. After 48 hours of incubation at 37°C, the filters (Millipore, USA) were taken out gently and cells on the upper surface of the filters were removed using cotton swabs. 1% of crystal violet (Genemed, USA) was used to stain the cells on the underside of Transwell filters for 10 minutes which were then photographed. Finally, the number of migrating cells stained with crystal violet was harvested and detected at 570 nm for quantitative assessment per filter.

### 2.8. Tube Formation Assay

To evaluate the effect of apigenin on angiogenesis, we performed tube formation assay. 50 *μ*L of ice-cold matrigel was added to 96-well plates. After OGD/R and/or treatment with apigenin, 2 × 10^4^/well HBMVECs were added in 200 *μ*L of media to the plates followed by incubation for 6 h, and the tube network was photographed. The tube networks were quantified using ImageJ software.

### 2.9. Apoptosis Assay

A cell apoptosis kit with Annexin V-APC and PI for flow cytometry (Sungene Biotech, China) was used to assay apoptosis of HBMVECs according to the manufacturer' instruction. In short, cells after OGD/R and treatment with apigenin were harvested and washed in ice-cold PBS and then double-stained with Annexin V-APC and PI. 5 × 10^4^ cells per sample were acquired in a BD FACSAria cell sorter flow cytometer, and the proportions of labeled cells were analyzed using BD Accuri C6 Software (Becton Dickinson).

### 2.10. Autophagy Assay

HBMVECs undergoing OGD/R and/or treatment with apigenin were fixed with 2.5% glutaraldehyde, and then fixed again with 1% osmium tetroxide (Sigma-Aldrich, MO, USA) for 30 minutes, followed by dehydration via acetone at room temperature, embedding via epoxy-embedding medium (Sigma-Aldrich, MO, USA), and ultrathin sections of 1 *μ*m were made and then stained by uranyl acetate (Tianfu Chemical Co. Ltd., China). Subsequently, the FEI Tecnai G2 Spirit transmission electron microscope (FEI, Netherlands) was used to observe the photograph.

### 2.11. Evaluating Apigenin Effect in Cerebral Ischemia/Reperfusion Injury Rats

To explore the effect of apigenin in cerebral ischemia/reperfusion injury in vivo, a rat middle cerebral artery occlusion (MCAO) model was established according to previous reports [19, 20]. The MCAO model was established using the line-embolus method in all model rats, and those in the sham operation group also underwent operation except for putting in the line-embolus. The line-embolus was taken out at 1.5 h postsuturing insertion to achieve middle cerebral artery (MCA) reperfusion (MACO/R). 117 healthy male Sprague-Dawley (SD) rats, weighing 250 ± 20 g, provided by the Experimental Animal Center of Wenzhou Medical University, were randomly divided into the sham operation group (*n* = 13), model group, model group treated with apigenin (*n* = 26), model group treated with Daidzin (Sigma-Aldrich, MO, USA) (*n* = 26), and model group treated with apigenin combined with Daidzin (*n* = 26). Dosage of apigenin was 25 mg/kg weight of rats and Daidzin was 0.4 mg/kg weight of rats every 24 hours post-MCAO operation via intraperitoneal injection. The model group was treated with equal amount of saline. 7 d and 14 d after MCAO/R, the Zea Longa 5-point system [21] was used to evaluate neurological symptoms of animals by two observers blinded to the treatment. Hematoxylin-eosin staining (HE) was performed for pathological examination of rat brains 7 d and 14 d post-MCAO/R and treatment.

7 d and 14 d after MCAO/R, rats were anesthetized with 100 mg/kg chloral hydrate and then subjected to intracardiac perfusion with ice saline. The head was removed quickly and the brain carefully dissected and sliced coronally at 2 mm spacing. The slices were then stained by 2% TTC buffer at 37°C for 30 minutes followed by being fixed in 4% paraformaldehyde overnight and the photograph being taken [22].

VEGFR2 and CD34 in the brain of rats at 7 d and 14 d after MCAO/R were detected by immunofluorescence staining. Alexa Fluor 647 goat anti-rabbit and FITC goat anti-rabbit antibodies were used for secondary antibodies. Nuclei were also counterstained with DAPI, and the stained cells were examined with fluorescence microscopy (TCS SP8 Leica, Germany). The sections were, respectively, stained with anti-VEGFR2 (Abcam, UK), anti-CD34 (Abcam, UK), and DAPI (Abcam, UK); the resulting images were then merged using Photoshop.

### 2.12. Data Analysis

Data are showed as means ± standard deviations (SD) of three independent samples. Student's unpaired *t*-test was used to evaluate the significant difference in two groups, and one-way ANOVA was involved in multiple comparisons. *P* value of 0.05 or less is considered statistically significant.

## 3. Result

### 3.1. Apigenin Inhibited Decrease of Cell Viability Induced by OGD/R through Regulation of Caveolin-1/VEGF

**Figure 1:**
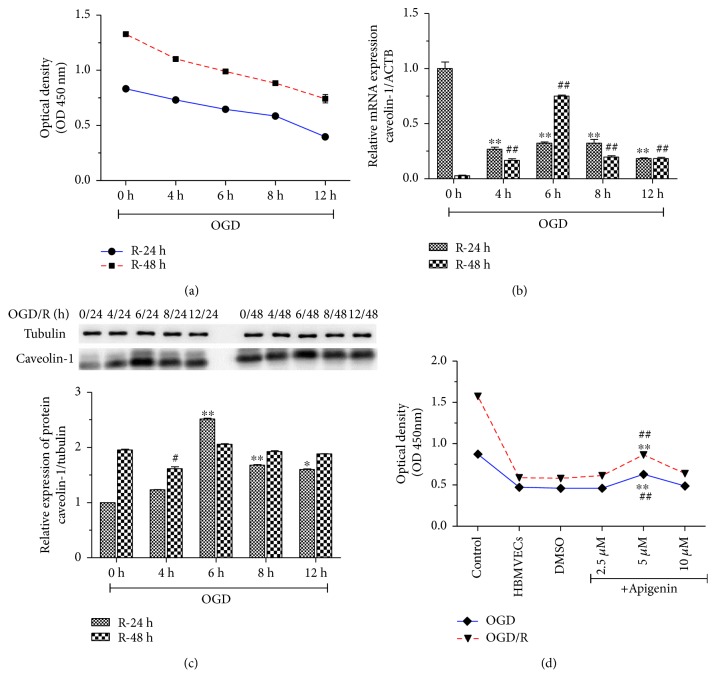
The effect of apigenin on cell viability of HBMVECs with OGD/R was explored. To make OGD/R, HBMVECs were cultured in conditions of oxygen deprivation, 1% O_2_ +  5% CO_2_ + 94% N_2_ for 4 h, 6 h, 8 h, and 12 h and glucose-free culture, then reoxygenated for 24 h or 48 h in a complete medium, and cell viability was measured using CCK8 (a), Caveolin-1 mRNA expression was detected by qPCR (b), and western blotting was used to assay Caveolin-1 protein expression (c). Cell viability of HBMVECs during OGD/R treated with 2.5, 5, and 10 *μ*M of apigenin were measured by CCK8 (d). Data was given as mean ± SD, *n* = 3. ^∗^*P* < 0.05 and ^∗∗^*P* < 0.01 compared with OGD; ^#^*P* < 0.05 and ^##^*P* < 0.01 compared with reoxygenation (R) in (a–c). ^∗^*P* < 0.05 and ^∗∗^*P* < 0.01 compared with the control group (normal culture); ^##^*P* < 0.01 compared with HBMVECs of OGD/R treated with DMSO in (d).

To investigate the effect of apigenin on cell viability, we first established the OGD/R model in HBMVECs. As shown in Figure 1(a), cell viability was decreased in a time-dependent manner during OGD and reperfusion. Expression of Caveolin-1 mRNA and protein was also influenced by OGD/R (Figures 1(b)–1(c)). OGD for 12 h and reperfusion for 24 h as cell viability was reduced to about 50% compared with normal cells were chosen for further study. Treatment of apigenin in a dose-dependent manner increased cell viability in the dose of 2.5 *μ*M to 5 *μ*M, and 10 *μ*M of apigenin inhibited cell viability compared with the OGD/R model group (*P* < 0.01) (Figure 1(d)). We chose 5 *μ*M of apigenin for the following cell experiments.

### 3.2. Apigenin Prompted Migration and Tube Formation of HBMVECs after OGD/R

**Figure 2:**
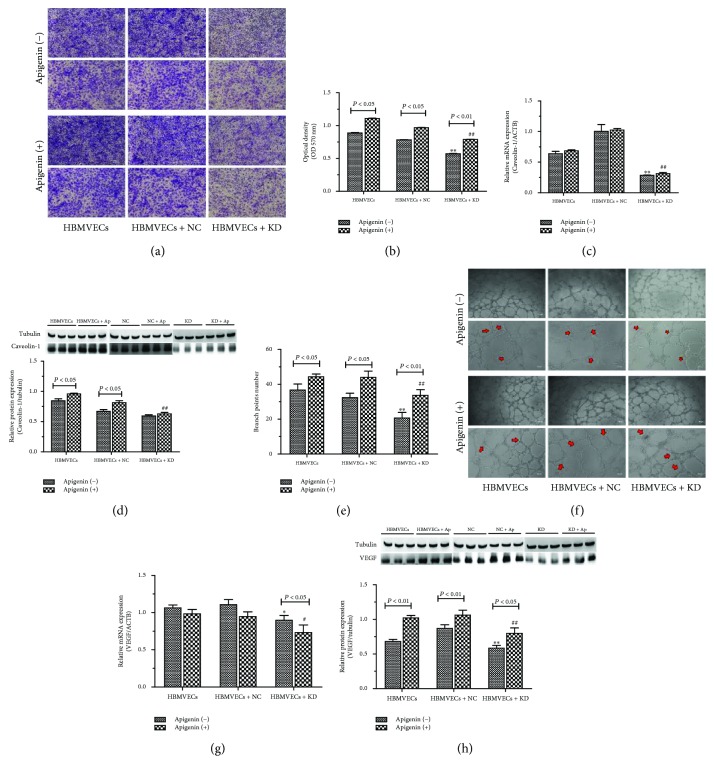
Apigenin affected cell migration and tube formation of HBMVECs with OGD/R. After OGD/R, lentivirus transfection, and/or treatment with apigenin to HBMVECs, cell migration was measured using the Transwell method (a), and the number of migrating cells stained with crystal violet was harvested and detected at 570 nm for quantitative assessment per filter (b); tube formation of cells was performed and tube networks were quantified as analysis branch points (red arrows indicated) for each field using ImageJ software (e–f). mRNA and protein expression of Caveolin-1 (c–d) and VEGF (g–h) were assayed by qPCR and western blotting, respectively. Data was given as mean ± SD, *n* = 3. ^∗∗^*P* < 0.01 compared with HBMVECs transfected by negative lentivirus (HBMVECs + NC) after OGD/R treated with solvent; ^#^*P* < 0.05 and ^##^*P* < 0.01 compared with HBMVECs + NC after OGD/R treated with apigenin. HBMVECs + KD means HBMVECs transfected by Caveolin-1 knockdown lentivirus.

Tube formation and migration assay were performed to investigate apigenin on neovascularization in vitro. Caveolin-1 involved in the mechanism of apigenin was also explored. Apigenin promoted cell migration significantly, and there was 24.7%, 24.2%, and 38.2% of migration cell increase in all the groups of HBMVECs, HBMVECs transfected with negative lentivirus (HBMVECs + NC), and Caveolin-1 knockdown lentivirus (HBMVECs + D) after OGD/R (*P* < 0.05 or *P* < 0.01) (Figures 2(a)–2(b)). Compared with the HBMVEC and HBMVEC + NC groups, cell migration ability of both apigenin- and solvent- (DMSO-) treated HBMVECs in the HBMVEC + KD group was significantly decreased (*P* < 0.01). To evaluate cell tube information ability, the branch point number was counted according to the Taylor et al. reports [23]. As shows in Figures 2(e)–2(f), cellular capacity of tube formation was significantly increased by apigenin in all the experimental groups. The number of tube formation was decreased approximately 21% in DMSO solvent-treated cells compared with apigenin-treated cells in HBMVEC and HBMVEC + NC groups, and apigenin prompted tube formation of about 35%. Compared with the HBMVEC + NC group, the number of tube formation was significantly decreased in the HBMVEC + KD group treated with apigenin and solvent (*P* < 0.05 or *P* < 0.01). Apigenin can promote but not reverse the inhibition effect of Caveolin-1 gene knockdown on the number of the tube formation and migration of HBMECs after OGD/R. It is noted in Figures 2(c)–2(d) that the mRNA and protein expression of Caveolin-1 in the HBMVEC + KD group was significantly decreased compared with that in the HBMVEC + NC group (*P* < 0.05 or *P* < 0.01) accompanied with VEGF protein expression decreasing (Figures 2(g)–2(h)). Apigenin significantly upregulated Caveolin-1 mRNA expression in the HBMVEC + KD group and protein expression of caveoloin-1 and VEGF increased in all the experiment groups. mRNA expression of VEGF was also found to be decreasing in apigenin-treated cells compared with solvent-treated ones in the HBMVEC + KD group (*P* < 0.05). Apigenin can promote the number of tube formation and migration of HBMECs, knockdown the Caveolin-1 gene, and inhibit angiogenesis. The apigenin's promoting effect does not reverse the inhibition effect of Caveolin-1 gene knockdown.

### 3.3. Apigenin Affected OGD/R-Induced Apoptosis and Autophagy of HBMVECs

**Figure 3:**
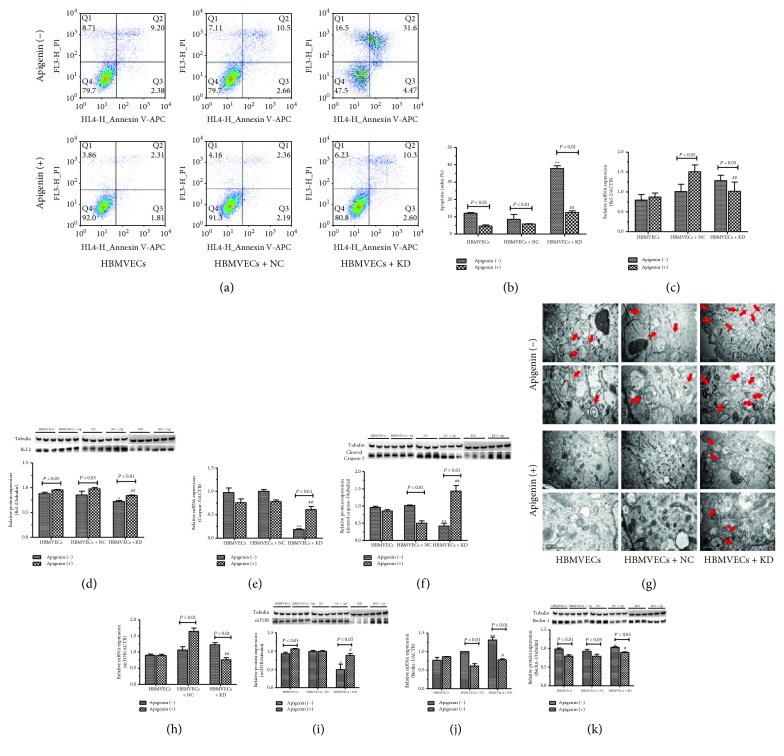
Apigenin inhibited apoptosis and autophagy of HBMVEC-combined OGD/R with Caveolin-1. Flow cytometer was used to measure apoptosis and transmission electron microscope to observe autophagy of OGD/R HBMVECs and/or treated with apigenin and intervened with by the Caveolin-1 lentivirus. The associated gene mRNA and protein expression was also assayed by qPCR and western blotting. (a–b) Results of apoptosis and analysis data are shown. mRNA expression of Bcl-2 and Caspase-3 are presented in (c) and (e), and protein expression are shown in (d) and (f). Photographs of cells were taken by transmission electron microscope system, and bilayer or multilayer autophagosome was indicated by red arrows (g). mTOR and Beclin-1 mRNA expression was measured (h, j), and protein expression was analyzed (i, k). Data was given as mean ± SD, *n* = 3. ^∗^*P* < 0.05 and ^∗∗^*P* < 0.01 compared with HBMVECs transfected by negative lentivirus (HBMVECs + NC) after OGD/R treated with solvent; ^#^*P* < 0.05 and ^##^*P* < 0.01 compared with HBMVECs + NC after OGD/R treated with apigenin. HBMVECs + KD means HBMVECs transfected by Caveolin-1 knockdown lentivirus.

Apoptosis of cells after OGD/R, lentivirus transfection, and treatment with apigenin were measured by flow cytometer.  Figure 3(a) shows that OGD/R induced HBMVEC apoptosis significantly with early rate of apoptosis being 8.5 ± 0.2% and late apoptosis rate being 9.5 ± 0.3%. The total apoptosis rate in cells treated with apigenin displayed 0.65-, 0.62-, and 0.63-folds lower than that in the solvent-treated group of Caveolin-1 knockdown (Caveolin-1-KD), transfected negative control lentivirus (NC), and untransfected (HBMVECs) cells. There was no significant difference of apoptosis in apigenin- and solvent-treated cells in between NC and HBMVECs. It is shown in Figure 3(b) that apoptosis in the Caveolin-1-KD group treated with apigenin or not was 1.52- and 1.73-folds higher than that of the corresponding cells in the NC group. The data of the qPCR assay demonstrated that mRNA of the Bcl-2 expression was significantly upregulated in the solvent-treated HBMVEC + KD group compared with the HBMVEC + NC group which was downregulated but not reversed by apigenin (Figure 3(c)). Apigenin prompted Bcl-2 protein expression in the HBMVEC and HBMVEC + NC groups while induced Caveolin-1-KD significantly decreased it (*P* < 0.05) (Figure 3(d)). As for the cleaved Caspase-3, mRNA expression was decreased but upregulated by apigenin treatment in the HBMVEC + KD group compared with the HBMVEC + NC group (Figure 3(e)), and protein expression in the apigenin-treated HBMVEC + NC group significantly decreased while it increased in the HBMVEC + KD group compared with the corresponding solvent-treated cells (Figure 3(f)). In the HBMVEC + NC group, cleaved Caspase-3 protein expression of apigenin-treated cells significantly decreased compared with solvent-treated ones.

A transmission electron microscope was used to observe apigenin affecting the autophagy of HBMVECs after OGD/R combined with Caveolin-1. It is shown in Figure 3(g) that OGD/R induced a bilayer or multilayer autophagosome which was further aggravated by Caveolin-1-KD. Apigenin inhibited autophagy in all the experiment groups. Autophagy-related mTOR and Beclin-1 and mRNA and protein expression were also measured by qPCR and western blotting. Compared with the HEMVEC + NC group, mRNA expression of mTOR in solvent-treated cells increased and in apigenin-treated cells decreased significantly while protein expression was opposite to mRNA expression in the HBMVEC + KD group (Figures 3(h)–3(i)). Apigenin decreased mTOR mRNA expression and increased protein expression but did not reverse the effect of Caveolin-1-KD. Beclin-1 mRNA expression in the apigenin-treated HBMVEC + KD group was higher than that in the HBMVEC + NC group; however the Beclin-1 mRNA expression in the solvent-treated group was significantly decreased (Figure 3(j)). Apigenin inhibited Beclin-1 protein expression in all the groups and did not reverse the effect of Caveolin-1-KD as the Beclin-1 protein expression was notably increased in the solvent- or apigenin-treated group compared with the HBMVEC + NC group (*P* < 0.05) (Figure 3(k)).

### 3.4. Apigenin Attenuated Brain Damage and Improved Neurological Function in Rats of MCAO/R

**Figure 4:**
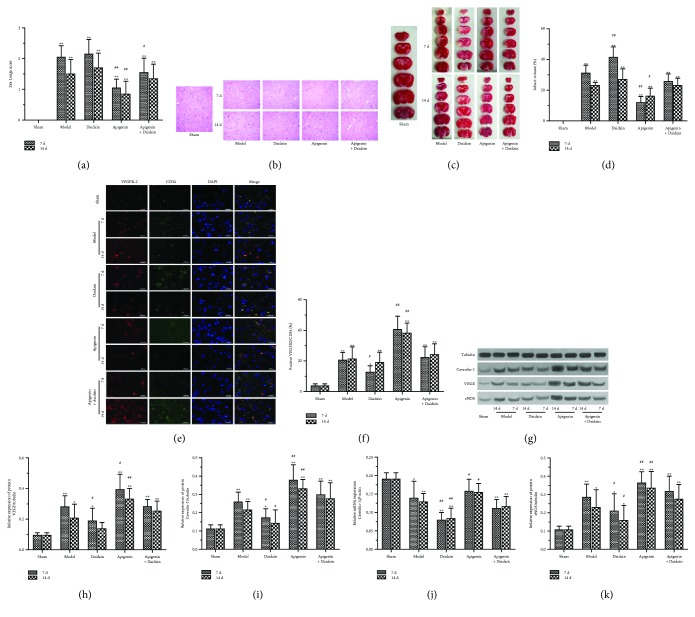
Effect of apigenin in rat MCAO/R model. Animals were divided into sham, middle cerebral artery occlusion/reperfusion (MCAO/R) (model), model rats treated with Caveolin-1 inhibitor of Daidzin (Daidzin), model rats treated with apigenin, and model rats treated with apigenin combined with Daidzin. Neurobehavioral scores, pathological examination, and measurement of Caveolin-1, VEGF, and eNOS were performed on the 7th and 14th days after anesthesia and ischemia/reperfusion in all groups. (a) Neurobehavioral scores by Zea Longa 5-point system were demonstrated. Results of hematoxylin-eosin staining of the brain tissue of rats in all the groups are shown in (b) and TTC staining results in (c) followed by analysis of infarct volume (d). VEGFR2/CD34 double-labeling endothelial progenitor cells (EPCs) were assayed using immunofluorescence (e), and VEGFR2/CD34 positive EPCs were analyzed (f). Caveolin-1 mRNA expression was detected by qPCR (j), and protein expression of Caveolin-1, VEGF, and eNOS was measured using western blotting (g, h, i, k). Data was given as mean ± SD, *n* = 6. ^∗^*P* < 0.05 and ^∗∗^*P* < 0.01 compared with rats in the sham group; ^#^*P* < 0.05 and ^##^*P* < 0.01 compared with rats in MCAO/R group.

We evaluated the effect of apigenin in the rat MCAO/R model. Animals were divided into the sham group and model group treated with placebo, apigenin, and/or Caveolin-1 inhibitor of Daidzin. Neurobehavioral scores were performed on the 7th and 14th days after anesthesia and ischemia/reperfusion in all groups. The neurobehavioral score was positive to the symptoms of neurological deficits in rats. The rats in the sham group displayed no neurological deficits and the ones in the model group had outstanding neurological deficits on their neurobehavioral scores which were significantly different from the sham group (*P* < 0.01) (Figure 4(a)). There were no significant differences of neurobehavioral scores between the Caveolin-1 inhibitor- (Daidzin-) treated and model (placebo) groups. Apigenin significantly reduced neurobehavioral scores compared with the placebo group. Daidzin attenuated the effect of apigenin. Neurological deficits of rats were significantly improved on day 14 (1.163 ± 0.655) after cerebral ischemia/reperfusion compared with 7 days (1.538 ± 0.670) (*P* < 0.05). Results of HE staining showed that no obvious pathological changes such as degeneration and necrosis of the nerve cells were found in sham group brain cells which were arranged neatly, cell structure was normal, the intercellular substance was even, and no edema appeared (Figure 4(b)). In the model group, different degrees of tissue cell damage, cytoplasmic edema, interstitial edema, widening of tissue gap and cells, deep staining, and cell shrinkage were observed in various degrees. It is also found that neuronal structures were fuzzy and cell bodies were swollen in cerebral ischemia penumbra areas. Compared with the placebo group, apigenin reduced the pathological changes of brain tissue at the two time points. The brain tissue edema at the two time points in the Daidzin combined with the apigenin intervention group was alleviated compared with only the Daidzin intervention group at the same time.

Results of TTC staining displayed that brain tissue in the sham group had no white infarction, all of which were red-stained. There were different areas of white infarctions mainly distributed in the middle cerebral artery blood supply areas as frontotemporal parietal cortex in all the model groups (Figure 4(c)). The cerebral infarction volume in the model group was significantly different from that in the sham group (*P* < 0.01) (Figure 4(d)). After cerebral Daidzin intervention, the volume of cerebral infarction was 25.89 ± 9.372 which was 19.75 ± 7.25 without Daidzin intervention. Caveolin-1 inhibitor intervention increased the volume of cerebral infarction (*P* < 0.05). At 7 days after cerebral ischemia, the volume of cerebral infarction in the apigenin intervention group was 19.26 ± 6.50 while the volume of cerebral infarction in the placebo intervention group was 29.80 ± 11.10; therefore, apigenin intervention reduced the volume of cerebral infarction (*P* < 0.05); at 14 days postblood, the effect of apigenin intervention on cerebral infarct volume was not significant (*P* > 0.05).

Endothelial progenitor cells (EPCs) are precursor cells of vascular endothelial cells, also known as angioblasts which participate in the formation of human embryonic angiogenesis and the repair process after endothelial injury [24]. It is considered currently that CD34 + EPC-labeled EPCs have stronger proangiogenic ability than other surface marker-type EPCs and have been widely used in the study of revascularization after cerebral ischemia. VEGFR2 is one of the most important receptors for VEGF which is mainly expressed in microvascular endothelial cells and endothelial progenitor cells. In our study, VEGFR2/CD34 immunofluorescence double-labeling was used as a marker for the proliferation of vascular endothelial cells. As shown in Figures 4(e)–4(f), there were few VEGFR2/CD34 double-labeled positive cells in the sham group, and different degrees of vascular endothelial neoplastic cells appeared in the treated model group, indicating that vascular regeneration can be induced after ischemic brain injury, which was consistent with previous reports [15]. Compared with that of the sham group, the number of VEGFR2/CD34 double-label positive cells in the cerebral ischemic penumbra of the model group increased significantly (*P* < 0.01). The number of VEGFR2/CD34 positive cells was 22.32 ± 10.12 after Caveolin-1 inhibitor intervention, and without caveolin-1 inhibitor intervention, the number of VEGFR2/CD34 positive cells was 29.43 ± 10.71. Caveolin-1 inhibitor intervention reduced the density of neovascularization (*P* < 0.01). The number of VEGFR2/CD34 positive cells was 31.08 ± 10.64 after apigenin intervention, and the number of VEGFR2/CD34 positive cells was 20.67 ± 8.64 without apigenin treatment. Apigenin intervention increased the density of neovascularization (*P* < 0.01). There was no significant difference in the cerebral ischemia time on neovascularization (*P* > 0.05).

The effect of apigenin on Caveolin-1, VEGF, and eNOS expression in the brain tissue of MCAO/R rats was investigated in the present study. The mRNA expression of Caveolin-1 decreased in rats after cerebral ischemia, and there was significant difference from the sham group (*P* < 0.05) (Figure 4(j)). Caveolin-1 mRNA expression after intervention with Daidzin was 0.097 ± 0.029, which was significantly lower than having no Daidzin intervention (*P* < 0.001). Apigenin intervention increased Caveolin-1 mRNA expression (*P* < 0.05), but did not reversed the effect of Daidzin completely. The time of cerebral ischemia had no significant effect on the expression of Caveolin-1 mRNA (*P* < 0.05). Compared with the sham group, Caveolin-1 protein expression significantly increased after cerebral ischemia in rats (*P* < 0.01) (Figure 4(i)). Daidzin intervention reduced Caveolin-1 protein expression (*P* < 0.01). Similar to mRNA expression, apigenin intervention increased Caveolin-1 protein level (*P* < 0.001), but did not reverse the effect of Daidzin. Caveolin-1 expression was higher at 7 d after cerebral ischemia than at 14 d in all the model groups (*P* < 0.05) (Figure 4(i)). Expression of VEGF and eNOS protein significantly increased in rats after cerebral ischemia compared with the sham group (*P* < 0.05). Daidzin downregulated VEGF and eNOS protein expression, and apigenin intervention increased VEGF and eNOS protein levels (*P* < 0.001), but did not reverse the effect of Daidzin, and the protein expression at 7 d was higher after cerebral ischemia than that at 14 d in all the model groups (*P* < 0.05), which was similar to the Caveolin-1 expression (Figures 4(h)–4(k)).

## 4. Discussion

Tissue damage by reperfusion injury, also called ischemia/reperfusion injury (IRI) or reoxygenation injury, is caused when blood supply returns to the tissue after a period of ischemia or lack of oxygen (hypoxia). IRI plays a part in the brain's ischemic cascade, which is involved in stroke and brain trauma. Stroke is the leading cause of disability and one of the leading causes of morbidity worldwide [20, 25]. It is reported that ischemia/reperfusion injury is involved directly in the potentiation of stroke damage [26]. Stroke syndromes most commonly affected the middle cerebral artery (MCA), and multiple methods of MCA occlusion (MCAO) have been described to mimic this clinical syndrome in animal models, and reperfusion after a period of occlusion has been included because recanalization commonly occurs following an acute stroke in the human [20]. Reperfusion of ischemic tissues is often associated with microvascular injury. Ischemic brain injury is a multifactor and multilevel complex pathological process based on cerebral vascular injury. The brain microvessels in ischemic brain injury are the early primary targets and important pathological events. The pathological damage triggers and aggravates the neuronal damage in the ischemic region. Therefore, it improves the postischemic cerebrovascular function and promotes cerebral vascular remodeling and promotes blood vessel regeneration.

Caveolin-1 is reported to regulate the blood-brain barrier (BBB) permeability and was downregulated, induced by nitric oxide (NO) in focal cerebral ischemia and reperfusion injury [27]. The vascular endothelial growth factor (VEGF) protected neurons from ischemic insults and promoted neurogenesis after cerebral ischemic injury because it has a crucial role in angiogenesis [14, 28, 29]. The two-way regulation of eNOS by Caveolin-1 plays an important role in angiogenesis [30]. Previous studies reported that Caveolin-1 gene ablation in mice could induce increased cerebral infarction volume and cell death, decreased VEGF expression, and impaired angiogenesis [29, 31]. Results of our previous study also indicated that upregulating the Caveolin-1/VEGF decreased cerebral infarct volume, promoted neurological recovery, and facilitated angiogenesis and neurogenesis [15, 29]. All the above studies suggested that Caveolin-1/VEGF is associated with functional recovery of cerebral ischemic stroke.

Apigenin is a natural and common dietary flavonoid found in fruits and vegetables [32] and has become an attractive compound in cancer research because of its antitumor properties against many human cancer cell lines, including lung [33], colon, breast [34, 35], colon [36], and thyroid [37]. Recently, it was reported that apigenin attenuated oxidative stress of erythrocytes [38], suppressed oxidative stress-mediated apoptosis to play a protective role on the rotenone-induced rat model of Parkinson's disease [39], and attenuated OGD/R-induced neuronal injury mainly through histone acetylation-mediated neurochemical alterations [18]. Apigenin could attenuate apoptosis in HEK293 cells by modulating the caspase signal pathway [40], and alleviate endotoxin-induced myocardial toxicity by modulating oxidative stress, autophagy, and inflammation [5], although apigenin could induce apoptosis and autophagy to play a role in chemoprevention in several human cancer cell lines [41].

Considering that Caveolin-1 has been reported to play an important role in angiogenesis after cerebral ischemic injury, to further understand the role and mechanism of Caveolin-1-mediated angiogenesis in the functional recovery of cerebral ischemic injury, and to investigate whether apigenin promotes angiogenesis after cerebral ischemic injury through the Caveolin-1 signaling pathway, we evaluated the effect of apigenin in vitro and in vivo. HBMVECs were used to make OGD/R in vitro. Lentivirus-mediated Caveolin-1 knockdown was performed in our study. Assay of cell proliferation, tuber formation and cell migration, apoptosis and autophagy, and apoptosis/autophagy-related genes as Bcl-2, cleaved Caspase-3, Beclin-1, and mTOR were carried out. Results indicated that apigenin prompted cell proliferation, tube formation and cell migration, while attenuating OGD/R-induced apoptosis and autophagy by affecting the expression of Caveolin-1/VEGF, Bcl-2, cleaved Caspase-3, Beclin-1, and mTOR. It was worth noting that a high dose of apigenin (10 *μ*M) showed cytotoxicity to HBMVECs, which may be similar to the previous study wherein apigenin has anticancer effects on cell proliferation and vascular endothelial growth factor (VEGF) transcriptional activation in a dose-dependent manner [33].

In vivo, Caveolin-1 was used as an entry point to establish a middle cerebral artery occlusion (MCAO) model in rats. Daidzin, as a Caveolin-1 inhibitor, was used to inhibit Caveolin-1 expression measured in the rat brain. Neurological function testing and pathological examination were performed on the 7th and 14th days after anesthesia and ischemia/reperfusion in our study. Expression of Caveolin-1, VEGF, eNOS, neovascularization, and apigenin interventions at different time points after ischemia/reperfusion was measured. The data demonstrated that apigenin could ameliorate neurological symptoms and reduce pathological changes and cerebral infarction volume, while increasing the number of VEGFR2/CD34 double-labeling positive EPCs which are used as a marker for proliferation of vascular endothelial cells in rats of MCAO/R. In vivo animal experiment results also indicated apigenin affected the Caveolin-1/VEGF pathway to attenuate cerebral infarction injury in rats of MCAO/R.

Considering our results that apigenin alleviated apoptosis and autophagy, promoted cell proliferation in HBMVECs of OGD/R and improved neovascularization, ameliorated neurological function, and reduced cerebral infarction volume to play an important role in protecting brain tissue from ischemia/reperfusion injury via the Caveolin-1/VEGF pathway, apigenin may provide a promising treatment for the brain against ischemia/reperfusion injury. Inadequacies of our study are that the protective effect of apigenin on cerebral ischemia/reperfusion injury is preliminary in vivo and some techniques of methods such as Evans blue + TTC staining, BBB permeability assessed by water content measure, and using clearer neurological methods to assess neurological severity should be performed in our further study.

## Figures and Tables

**Table 1:** Primers used in the qPCR assay.

Gene	Primer	Primer sequence (5′–3′)	Product
ACTB	Forward	CCCATCTATGAGGGTTACGC	150 bp
	Reverse	TTTAATGTCACGCACGATTTC	

Caveolin-1	Forward	CCCCAAGCATCTCAACGAC	151 bp
	Reverse	GGTAGACAGCAAGCGGTAAAAC	

VEGF	Forward	GCAGAAGGAGGAGGGCAG	90 bp
	Reverse	CACCAGGGTCTCGATTGGAT	

Bcl-2	Forward	CTTCGCCGAGATGTCCAGC	100 bp
	Reverse	CCAGTTCACCCCGTCCCT	

Caspase-3	Forward	CTGGAATGACATCTCGGTCTG	273 bp
	Reverse	TTCTACAACGATCCCCTCTGA	

Beclin-1	Forward	ACCGCAAGATAGTGGCAGAA	262 bp
	Reverse	CCCAGCCTGAAGTTATTGATTG	

mTOR	Forward	CAGGGGACACTTTTACCGC	252 bp
	Reverse	TGAGAATCAGACAGGCACGAA	

## Data Availability

The data used to support the findings of this study are included within the article.

## Abbreviations

HBMVECs: Human brain microvascular endothelial cells
OGD/R: Oxygen-glucose deprivation/reoxygenation
DMSO: Dimethyl sulfoxide
VEGFR2: Vascular endothelial growth factor receptor 2
TTC: 2,3,5-Triphenyltetrazolium chloride
IHC: Immunohistochemistry
MCAO: Middle cerebral artery occlusion
VEGF: Vascular endothelial growth factor
HE: Hematoxylin-eosin staining
EPCs: Endothelial progenitor cells
eNOS: Endothelial nitric oxide synthase.

## References

[1] Kalogeris T., Baines C. P., Krenz M., Korthuis R. J. (2016). Ischemia/reperfusion. *Comprehensive Physiology*.

[2] Lee J. M., Grabb M. C., Zipfel G. J., Choi D. W. (2000). Brain tissue responses to ischemia. *The Journal of Clinical Investigation*.

[3] Kristian T. (2004). Metabolic stages, mitochondria and calcium in hypoxic/ischemic brain damage. *Cell Calcium*.

[4] Visioli F., Bellomo G., Galli C. (1998). Free radical-scavenging properties of olive oil polyphenols. *Biochemical and Biophysical Research Communications*.

[5] Li F., Lang F., Zhang H. (2017). Apigenin alleviates endotoxin-induced myocardial toxicity by modulating inflammation, oxidative stress, and autophagy. *Oxidative Medicine and Cellular Longevity*.

[6] Dang Y., Li Z., Luo B., Pan L., Wei Q., Zhang Y. (2017). Protective effects of apigenin against acrylonitrile-induced subchronic sperm injury in rats. *Food and Chemical Toxicology*.

[7] Liu Y., Wang L., Du Y. (2017). Effects of apigenin pretreatment against renal ischemia/reperfusion injury via activation of the JAK2/STAT3 pathway. *Biomedicine & Pharmacotherapy*.

[8] Siddique Y. H., Jyoti S. (2017). Alteration in biochemical parameters in the brain of transgenic Drosophila melanogaster model of Parkinson’s disease exposed to apigenin. *Integrative Medicine Research*.

[9] Liu P., Rudick M., Anderson R. G. W. (2002). Multiple functions of caveolin-1. *The Journal of Biological Chemistry*.

[10] Navarro A., Anand-Apte B., Parat M. O. (2004). A role for caveolae in cell migration. *The FASEB Journal*.

[11] Zhang Z., Chopp M. (2002). Vascular endothelial growth factor and angiopoietins in focal cerebral ischemia. *Trends in Cardiovascular Medicine*.

[12] Ma Y., Zechariah A., Qu Y., Hermann D. M. (2012). Effects of vascular endothelial growth factor in ischemic stroke. *Journal of Neuroscience Research*.

[13] Pignataro G., Ziaco B., Tortiglione A. (2015). Neuroprotective effect of VEGF-mimetic peptide QK in experimental brain ischemia induced in rat by middle cerebral artery occlusion. *ACS Chemical Neuroscience*.

[14] Hansen T. M., Moss A. J., Brindle N. P. (2008). Vascular endothelial growth factor and angiopoietins in neurovascular regeneration and protection following stroke. *Current Neurovascular Research*.

[15] Gao Y., Zhao Y., Pan J. (2014). Treadmill exercise promotes angiogenesis in the ischemic penumbra of rat brains through caveolin-1/VEGF signaling pathways. *Brain Research*.

[16] Zhao Y., Pang Q., Liu M. (2017). Treadmill exercise promotes neurogenesis in ischemic rat brains via caveolin-1/VEGF signaling pathways. *Neurochemical Research*.

[17] Pang Q., Zhang H., Chen Z. (2017). Role of caveolin-1/vascular endothelial growth factor pathway in basic fibroblast growth factor-induced angiogenesis and neurogenesis after treadmill training following focal cerebral ischemia in rats. *Brain Research*.

[18] Tu F., Pang Q., Huang T., Zhao Y., Liu M., Chen X. (2017). Apigenin ameliorates post-stroke cognitive deficits in rats through histone acetylation-mediated neurochemical alterations. *Medical Science Monitor*.

[19] Liu L., Cen J., Man Y. (2018). Transplantation of human umbilical cord blood mononuclear cells attenuated ischemic injury in MCAO rats via inhibition of NF-*κ*B and NLRP3 inflammasome. *Neuroscience*.

[20] Uluc K., Miranpuri A., Kujoth G. C., Akture E., Baskaya M. K. (2011). Focal cerebral ischemia model by endovascular suture occlusion of the middle cerebral artery in the rat. *Journal of Visualized Experiments*.

[21] Longa E. Z., Weinstein P. R., Carlson S., Cummins R. (1989). Reversible middle cerebral artery occlusion without craniectomy in rats. *Stroke*.

[22] Meng Y.-C., Ding Z.-Y., Wang H.-Q., Ning L.-P., Wang C. (2015). Effect of microRNA-155 on angiogenesis after cerebral infarction of rats through AT1R/VEGFR2 pathway. *Asian Pacific Journal of Tropical Medicine*.

[23] Taylor A. C., Seltz L. M., Yates P. A., Peirce S. M. (2010). Chronic whole-body hypoxia induces intussusceptive angiogenesis and microvascular remodeling in the mouse retina. *Microvascular Research*.

[24] Asahara T., Murohara T., Sullivan A. (1997). Isolation of putative progenitor endothelial cells for angiogenesis. *Science*.

[25] Nour M., Scalzo F., Liebeskind D. S. (2013). Ischemia-reperfusion injury in stroke. *Interventional Neurology*.

[26] Denner L. (2001). Stroke and ischemia-reperfusion injury. *IDrugs*.

[27] Gu Y., Zheng G., Xu M. (2012). Caveolin-1 regulates nitric oxide-mediated matrix metalloproteinases activity and blood-brain barrier permeability in focal cerebral ischemia and reperfusion injury. *Journal of Neurochemistry*.

[28] Zarbin M. A. (2018). Anti-VEGF agents and the risk of arteriothrombotic events. *Asia-Pacific Journal of Ophthalmology*.

[29] Liu M., Wu Y., Liu Y. (2018). Basic fibroblast growth factor protects astrocytes against ischemia/reperfusion injury by upregulating the caveolin-1/VEGF signaling pathway. *Journal of Molecular Neuroscience*.

[30] Chen Z., Oliveira S. D. S., Zimnicka A. M. (2018). Reciprocal regulation of eNOS and caveolin-1 functions in endothelial cells. *Molecular Biology of the Cell*.

[31] Jasmin J. F., Malhotra S., Singh Dhallu M., Mercier I., Rosenbaum D. M., Lisanti M. P. (2007). Caveolin-1 deficiency increases cerebral ischemic injury. *Circulation Research*.

[32] Dunnick J. K., Hailey J. R. (1992). Toxicity and carcinogenicity studies of quercetin, a natural component of foods. *Fundamental and Applied Toxicology*.

[33] Liu L. Z., Fang J., Zhou Q., Hu X., Shi X., Jiang B. H. (2005). Apigenin inhibits expression of vascular endothelial growth factor and angiogenesis in human lung cancer cells: implication of chemoprevention of lung cancer. *Molecular Pharmacology*.

[34] Yin F., Giuliano A. E., Law R. E., Van Herle A. J. (2001). Apigenin inhibits growth and induces G2/M arrest by modulating cyclin-CDK regulators and ERK MAP kinase activation in breast carcinoma cells. *Anticancer Research*.

[35] Vrhovac Madunic I., Madunic J., Antunovic M. (2018). Apigenin, a dietary flavonoid, induces apoptosis, DNA damage, and oxidative stress in human breast cancer MCF-7 and MDA MB-231 cells. *Naunyn-Schmiedeberg's Archives of Pharmacology*.

[36] Wang W., VanAlstyne P. C., Irons K. A., Chen S., Stewart J. W., Birt D. F. (2004). Individual and interactive effects of apigenin analogs on G2/M cell-cycle arrest in human colon carcinoma cell lines. *Nutrition and Cancer*.

[37] Banerjee K., Mandal M. (2015). Oxidative stress triggered by naturally occurring flavone apigenin results in senescence and chemotherapeutic effect in human colorectal cancer cells. *Redox Biology*.

[38] An F., Cao X., Qu H., Wang S. (2015). Attenuation of oxidative stress of erythrocytes by the plant-derived flavonoids vitexin and apigenin. *Pharmazie*.

[39] Anusha C., Sumathi T., Joseph L. D. (2017). Protective role of apigenin on rotenone induced rat model of Parkinson’s disease: suppression of neuroinflammation and oxidative stress mediated apoptosis. *Chemico-Biological Interactions*.

[40] Zhong Y., Jin C., Gan J. (2017). Apigenin attenuates patulin-induced apoptosis in HEK293 cells by modulating ROS-mediated mitochondrial dysfunction and caspase signal pathway. *Toxicon*.

[41] Sung B., Chung H. Y., Kim N. D. (2016). Role of apigenin in cancer prevention via the induction of apoptosis and autophagy. *Journal of Cancer Prevention*.

